# Neural Surface Antibodies and Neurodegeneration: Clinical Commonalities and Pathophysiological Relationships

**DOI:** 10.3390/biomedicines11030666

**Published:** 2023-02-22

**Authors:** Maria Pia Giannoccaro, Federico Verde, Luana Morelli, Giovanni Rizzo, Fortuna Ricciardiello, Rocco Liguori

**Affiliations:** 1 IRCCS Istituto delle Scienze Neurologiche di Bologna, 40139 Bologna, Italy; 2 Dipartimento di Scienze Biomediche e Neuromotorie, Università di Bologna, 40126 Bologna, Italy; 3 Department of Neurology and Laboratory of Neuroscience, IRCCS Istituto Auxologico Italiano, 20149 Milan, Italy; 4 Department of Pathophysiology and Transplantation, “Dino Ferrari” Center, Università degli Studi di Milano, 20122 Milan, Italy

**Keywords:** neuronal surface antigens, antibodies, neurodegeneration, autoimmune encephalitis, biomarkers

## Abstract

Autoimmune encephalitis and neurodegenerative disorders share several clinical features, including behavioural and psychiatric manifestations, cognitive impairment, sleep and movement disorders. Therefore, it is not surprising that autoimmune encephalitis is one of the main differential diagnoses of rapidly progressive dementia. However, more chronic presentations of autoimmune disorders have been reported and can lead to the misdiagnosis of a neurodegenerative disease. On the other hand, antibodies against neuronal proteins, such as those directed against NMDAR, can occur during established neurogenerative disorders, and their role in this context is still unclear. They might be simple bystanders or modify the disease course and phenotype. Indeed, autoimmune encephalitis can leave long-term cognitive sequelae and specific antibodies to neuronal surface antigens are associated with clinical and pathological neurodegenerative features. Here we review the link between these antibodies and neurodegeneration. In particular we discuss: (a) the possibility that autoimmune encephalitis presents as a neurodegenerative disease, identifying the red flags that can help in the differential diagnosis between antibody-mediated and neurodegenerative disorders; (b) the occurrence of antibodies against neuronal surface antigens in patients with neurodegenerative disorders and their possible role in the disease course; and (c) the long-term cognitive and neuroradiological changes associated with autoimmune encephalitis, as well as the biomarkers that can help to predict the cognitive outcome. Finally, we review the clinical and pathological features of IgLON5 antibodies-related encephalitis, a unique model of the relationship between antibodies and neurodegeneration.

## 1. Introduction

Autoimmune encephalitis (AE) represents a group of brain disorders characterised by the subacute onset of diverse neurological and neuropsychiatric symptoms due to a pathologic immune response directed against self-antigens expressed in the central nervous system. AE is often associated with antibodies against neuronal surface antigens (NSA-Ab) [1,2]. Due to the wide and different expression of the various antigenic targets, clinical manifestations are variable and include diverse combinations of cognitive dysfunction, psychiatric and behavioural disturbances, movement disorders, seizures, sleep disorders and dysautonomia, among others (Table 1) [2].

Many NSA-Ab-related syndromes are associated with cognitive impairment and sometimes this is the first and prevalent manifestation, challenging the diagnosis. This is particularly true in the elderly, as encephalitic signs can be absent [3], and the disease can progress slowly, mimicking a primary neurodegenerative disorder.

On the other hand, the idea that autoantibodies could be involved in dementia is old [4,5,6] and several targets have been investigated over time including neuronal receptors and glial protein (for review see [7]). Antibodies against several neuronal proteins involved in different neurodegenerative diseases (i.e., amyloid β (Aβ), tau and alpha-synuclein) occur in both healthy subjects and in pathological conditions and they could have either a physiological role in preventing the accumulation of pathological proteins or exert additional damage [8]. Antibodies against neuronal proteins might therefore be part of the homeostatic immunity, emerging for example secondarily to the physiological turnover of neurons outside the CNS, such as in the enteric nervous system [9]. After the discovery of NMDAR-Ab in patients with NMNDAR encephalitis [10], the presence of these antibodies was investigated in a few cohorts of patients with neurodegenerative disorders [11,12,13]. Oppositely to what is generally observed in patients with NSA-Ab-related AE, these antibodies are often found only in the serum in patients without encephalitic signs and often belong to the IgA/IgM subclasses (instead of IgG) [11,12,13,14,15], and therefore their role is not yet clear. In this context, NSA-Ab could be bystanders of a degenerative process or play a pathogenic function, if not primary at least secondary, acting as modifiers of the clinical phenotype and of the disease course. Indeed, NSA-Ab have the potential to cause secondary neurodegeneration even in typical AE, with mechanisms that have not yet been fully elucidated.

**Table 1:** The main clinical features and pathogenic mechanisms of NSA-Ab.

Antibody Target	Age/ Sex Prevalence	Clinical Syndromes/Symptoms	Acute Phase Cognitive Features	Ancillary Test	Antibody Pathogenicity and Mechanism of Cognitive Impairment
NMDAR [10,16,17,18]	Young/F	Encephalitis with psychiatric symptoms, seizures, movement disorders, autonomic instability, hypoventilation, coma	Severe cognitive dysfunction involving all domains (memory, executive function, attention, language, visuospatial processing and social cognition)	MRI: mainly normal (70–80%) or unspecific. CSF: 80% abnormal (pleocytosis, oligoclonal bands); EEG: usually abnormal (90%) with slow wave or epileptic changes and rare delta brush	NMDAR-Ab cause cross-linking and internalization with reduced NMDARs density and consequent reduced NMDAR-dependent LTP leading to memory dysfunction
LGI1 [19,20,21,22,23]	Elderly/M	Limbic encephalitis with memory impairment and seizures. FBDS can precede the onset of cognitive impairment	Disorientation, confusion and autobiographical memory impairment	MRI: abnormal (75%), mostly showing increased medial temporal lobe and hippocampus signal; CSF: abnormal 25% (pleocytosis and increased proteins); EEG: 50% abnormal (epileptiform activity or slowing)	Disruption of the interaction with ADAM22 and reduction of AMPAR; possible complement activation. Antibodies abrogate LTP induction at CA3-CA1 hippocampal synapses in animal model leading to memory impairment
CASPR2 [19,24,25,26,27]	Elderly/M	Limbic encephalitis, neuromyotonia and Morvan’s syndrome, neuropathic pain	Common cognitive dysfunction, anterograde and episodic memory impairment	MRI: more often normal (30% abnormal signals in the hippocampus); CSF: often normal (30% pleocytosis, increased proteins or oligoclonal bands); EEG: 70% abnormal (epileptic changes or slowing)	CAPSR2 internalization and inhibition of CASPR2/TAG1 interaction. Possible reduction of Kv1 and AMPARs leading to neuronal hyperexcitability and ineffective recruitment of post-synaptic AMPARs leading to memory impairment
GABAB receptor [28,29,30,31]	Elderly/M	Limbic encephalitis, seizures	Cognitive/behavioural dysfunction in most patients with memory impairment and confusion	MRI: abnormal signals in the temporal lobe and hippocampus (70%); CSF: frequently altered (80%); EEG: epileptic changes (80%)	Antibodies can prevent the activation of the GABABR and block its function; however, how this mechanism can impact memory has not been investigated in vivo
AMPA receptor [32,33,34,35,36]	Middle aged/F	Limbic encephalitis, seizures	Prominent memory impairment: amnesia could be isolated at onset	MRI: often bilateral medial temporal lobes and insular cortex involvement; CSF: 70% abnormal; EEG: abnormal in about half cases	Internalisation of GluA2 containing AMPARs with reduction of extrasynaptic AMPAR leading to LTP changes and consequent memory impairment
GABAA receptor [37,38]	Broad age range/no sex predominance	Encephalitis and refractory seizures or status epilepticus	Cognitive impairment in two thirds of patients	MRI: multifocal cortical and subcortical FLAIR signal abnormalities: CSF. Abnormal in about 50% of cases; EEG: usually abnormal	Antibodies selectively remove GABAAR from synapses, downregulation of the GABAAR function; however how this mechanism can impact cognition has not been investigated in vivo
DPPX [39,40]	Middle aged/M	Weight loss/diarrhoea, cognitive/behavioural changes, CNS hyperexcitability (myoclonus, tremors and hyperekplexia)	Memory loss, confusion	MRI: normal or unspecific; CSF: often normal; EEG: 70% abnormal	DPPX and Kv4.2 membrane expression is reduced by patient antibodies, whilst enteric neuron activity is increased; how this mechanism can impact cognition has not been investigated in vivo
Glycine receptor [41,42,43,44]	Broad age range/M	Progressive encephalopathy with rigidity and myoclonus (PERM), epilepsy, SPS	Cognitive deficits, encephalopathy	MRI: often normal or non-specific; CSF: abnormal in less than half cases; EEG: often abnormal (70%), with focal/diffuse slowing or rarer epileptic changes	GlyR internalisation; how this mechanism can impact cognition has not been investigated in vivo
IgLON5 [45,46,47]	Elderly/no sex predominance	Sleep disorder, bulbar symptoms, gait abnormality followed by cognitive dysfunction	Sleep disorder, bulbar symptoms and gait abnormality followed by cognitive dysfunction	MRI: often normal or non-specific; CSF: 60% pleocytosis	Internalisation with reduction of IgLon5 expression (irreversible in the long term after the removal of antibodies); memory impairment due to gamma-oscillation dysfunction, neurodegeneration and synaptic dysfunction

CSF: cerebrospinal fluid; EEG: electroencephalogram; F: female; M: male; MRI: magnetic resonance imaging.

Finally, in the last years, the borders between autoimmunity and neurodegeneration have been further thinned by the discovery of IgLON5 antibodies [45], which has shed new light on the possible role of autoimmunity in neurodegenerative disorders.

**Figure 1:**
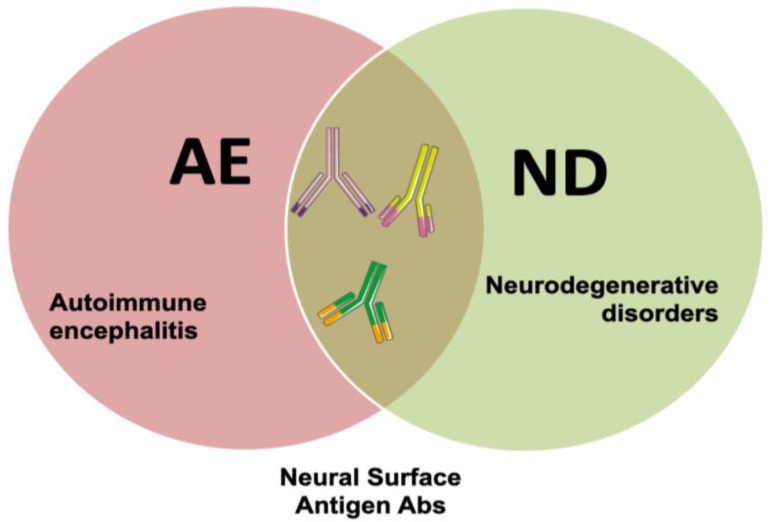
Interplay between AE and neurodegeneration mediated by NSA-Ab. AE can present with neurodegenerative-like phenotype and NSA-Ab can cause neurodegeneration in patients with AE. On the other hand, NSA-Ab might be found in patients with neurodegenerative disorders, although their significance is still unclear.

Others have reviewed the role of antibodies directed against neuronal proteins involved in different neurodegenerative diseases (e.g., amyloid β (Aβ)) in the pathogenesis of these disorders [8]. Here, we focus specifically on antibodies against neuronal surface antigens primarily involved in AE, reviewing the different interplay between neuronal surface antibody-mediated autoimmunity and neurodegeneration (Figure 1). We first discuss the presentation of AE as a neurodegenerative-like disorder and the red flags that could help in the differential diagnosis between these conditions. Secondly, we present the current evidence of the occurrence and possible role of NSA-Ab in neurodegenerative diseases. We further highlight the long-term neuropsychological, neuroimaging and biomarker changes associated with NSA-Ab in AE patients and discuss the possible underlying mechanisms leading to neurodegeneration in this context. Finally, we present the current knowledge on IgLON5 disease as a new model of interaction between autoimmunity and neurodegeneration.

## 2. Neuronal Surface Antibody-Mediated Encephalitis Presenting as a Neurodegenerative Disorder

AE and neurodegenerative disorders share several features including behavioural and psychiatric disorders, cognitive impairment, movement and sleep disorders. 

Patients with AE usually present a combination of clinical features associated with variable brain MRI and CSF inflammatory changes (Table 1). However, cognitive impairment and/or movement and sleep disorders can be the main clinical feature misleading the diagnosis towards a neurodegenerative disorder. It is not surprising that AEs are increasingly recognised as one of the most common causes of treatable rapid progressive dementias, with frequencies progressively increasing over time thanks also to the expanding list of NSA-Ab [48,49,50,51,52]. 

In a setting when an autoimmune aetiology for the dementia is strongly suspected, Flanagan et al. [53] reported the presence of autoantibodies targeting the VGKC complex in 15% of cases. In the same cohort, 64% of cases responded to immunotherapy. A shorter delay from symptom onset to initiation of therapy for autoimmune dementia increased the likelihood of response [53]. However, 41% of immunotherapy-responsive dementia patients had normal brain MRIs, and many patients showed normal CSF and EEG; almost 9% of the disorders had been initially diagnosed as Creutzfeldt-Jakob disease (CJD) [53]. Several other reports described patients with rapidly progressive dementia and antibody mediated encephalitis [28,54,55].

However, not all patients with antibody-mediated dementia present with a rapidly evolving clinical picture. Autoimmune-mediated cognitive decline can progress slowly over many months, and therefore may be mistaken for a primary neurodegenerative disorder such as Alzheimer’s disease (AD) or frontotemporal dementia (FTD) [48,56]. NMDAR encephalitis might present with atypical features and prominent cognitive disturbances, including memory loss, cognitive fluctuations, visual hallucinations and sleep disorder, reminiscent of Lewy Body Dementia (LBD) [57]. Similarly, due to the prominent memory impairment, often associated with confusion and disorientation, LGI1 encephalitis could be misdiagnosed as neurodegenerative dementia including AD and LBD [58,59,60]. In a recent retrospective study, exploring the frequency of dementia diagnosis in patients over the age of 45 with LGI, NMDAR, CASPR2 and GABABR encephalitis, 67/175 (38%) were found to satisfy the 2011 NINCDS-ADRDA criteria for dementia and in 33 (52%) a neurodegenerative disorder was suspected [61]. Interestingly, imaging or CSF inflammatory signs were absent in 25% of patients and in 61% of 44 patients in whom neurodegenerative CSF biomarkers were assessed, at least one was found altered [61], underlying the difficulties of differential diagnosis between AE and dementia in certain clinical contexts. 

However, not only dementias but also other neurodegenerative diseases, mainly presenting with movement disorders, can be associated with the presence of NSA-Ab. Patients with LGI1, IgLON5, DPPX and GABABR antibodies have been misdiagnosed with Parkinson’s disease (PD), progressive supranuclear palsy (PSP), cortico-basal syndrome (CBS) or multisystem atrophy (MSA) [45,62,63,64,65]. REM sleep behaviour disorder (RBD), which can anticipate by decades the onset of a neurodegenerative disease, in particular MSA and PD [66], has been reported in patients with CASPR2 and LGI1-antibody-associated limbic encephalitis [19,67,68] and with IgLON5-antibody linked neurodegeneration [45]. Finally, status dissociatus, a complete breakdown of the boundaries of the different states of being, which are wakefulness, REM sleep, and non-REM sleep, with motor hyperactivity, which may be observed in the final phase of several different neurodegenerative disorders, has also been reported in patients with CASPR2-Abs, and less commonly, LGI1-Abs or NMDAR-Abs [69,70], widening the overlap between neurodegenerative and autoimmune conditions. 

We explored the frequency of NSA-Ab in patients who had received a diagnosis of a wide range of neurodegenerative disorders. We observed NSA-Ab in 13.8% (13/93) of patients vs. 2% of controls, with no difference between patients presenting with prominent dementia or parkinsonism [71]. When we retrospectively applied the AE criteria [1] in the seropositive cases without including the antibody results, five patients did not meet the criteria for possible AE because they had a chronic course, whereas in three cases it was not possible to exclude other diagnoses. Of the remaining five patients, who met criteria for possible AE, only two showed inflammatory CSF changes and none had AE-like MRI abnormalities [71]. Altogether these observations underline on one hand, the difficulty of differentiating AE from neurodegenerative disorders in the setting of a slowly evolving clinical picture, on the other hand they support the possibility that secondary NSA-Ab might develop during the course of neurodegenerative disorders, as discussed below.

### Diagnostic Clues to the Autoimmune Aetiology

Given the fact that AE and “autoimmune dementia” are potentially treatable disorders, it is important to achieve an early diagnosis. On the other hand, the indiscriminate search of NSA-Ab could lead to misdiagnosis and overtreatment along with over-utilization of resources. The need to identify patients with AE early led to the creation of consensus criteria [1], mainly based on the clinical features (i.e., subacute onset of cognitive impairment, behavioural and psychiatric changes and focal CNS signs) and brain MRI (i.e., hyperintense T2/FLAIR signal involving one or both medial temporal lobes, or in multifocal areas involving grey matter, white matter, or both) and CSF findings suggestive of neuroinflammation (i.e., pleocytosis and oligoclonal bands) or epileptic or slow-wave activity involving the temporal lobes on the EEG [1]. In this setting, the differential diagnosis of AE from even rapidly progressive dementia should be relatively easy. Moreover, patients with neurodegenerative dementia should have additional supporting findings such as specific changes in CSF neurodegeneration markers (i.e., altered Aβ42, total tau and phosphorylated tau values and ratios) [72] or molecular imaging specific alterations that should be absent in patients with AE [73]. However, in clinical practice this distinction is not always straightforward. Indeed, antibody-mediated cognitive deficits can evolve slowly over time, and inflammatory changes can be absent, particularly in the elderly population and in association with certain antibodies [3]. On the other hand, a tumultuous evolution of cognitive deficits and a frankly inflammatory CSF can be observed also in patients with primary degenerative disorders [74,75].

To help clinicians to promptly recognize patients with potentially treatable disorders, several studies tried to identify the main features associated with an “autoimmune dementia”. These have been classically defined as: (1) subacute onset with a rapidly progressive course; (2) symptoms fluctuation; (3) presence of tremor/myoclonus; (3) coexisting organ-specific autoimmunity; (4) inflammatory CSF; (5) presence of MRI changes suggestive of an inflammatory process; (6) risk factors or recent history of tumour [48,53,56,76]. A more recent study suggested that the presence of subtle seizures could be a red flag for AE in patients with dementia [61]. Nevertheless, seizures can occur also in neurodegenerative disorders [77], even early in the disease course [78], therefore the results of other investigations including imaging features and pathological biomarkers should be considered in the differential diagnosis. Nevertheless, in the same study, as already mentioned, 14 out of 44 patients tested had a CSF biomarker profile suggestive of AD or CJD, challenging the differential diagnosis [61]. In these cases, more specific tests such as RT-QuIC can help to exclude CJD, whereas an increased IgG index might suggest an autoimmune aetiology. We found that an irregular progression and the lack of a diagnosis for a specific type of dementia or parkinsonism associate with the presence of NSA-Ab [71]. However, the utility of these suggested red-flags in guiding the antibody testing has not been systematically assessed in prospective studies. Dubey and colleagues recently evaluated the diagnostic performance of the antibody-prevalence-in-epilepsy and encephalopathy (APE^2^) and responsive-to-immunotherapy-in-epilepsy and encephalopathy (RITE^2^) scores in the evaluation and management of patients with encephalopathy and cognitive impairment [79]. They observed that an APE^2^ score ≥ 4 was 99% sensitive and 93% specific for neural-specific-antibodies and that a RITE^2^ score ≥ 7 had 96% sensitivity and 86% specificity for favourable initial immunotherapy response supporting the utility of this approach in selecting patients candidate to antibody screening. However, these results warrant replication in prospective cohorts.

## 3. NSA-Ab in Patients with Neurodegenerative Disorders

Despite the clear existence of cases of immunotherapy-responsive dementia associated with NSA-Ab, the presence of several neuronal antibodies has been reported in patients with ascertained prion disease [80,81,82,83,84,85], challenging the significance of this finding and further complicating this diagnostic dilemma in cases with more chronic presentations. However, it is likely that these findings reflect the inclusion of patients with other diagnoses or a secondary phenomenon. Indeed, the frequency of NSA-Ab appears rare in patients with pathologically confirmed CJD [84,86].

To explore the prevalence of NSA-Ab in patients with defined primary dementias, Çoban A et al. [87] investigated 50 patients, finding NMDAR antibodies in one case presenting with LBD phenotype. Even though this patient had some features suggestive of an autoimmune aetiology, the presence of “atypical features” in the remaining cases failed to predict the presence of an NSA-Ab, highlighting the necessity to identify other markers predictive of an autoimmune dementia. However, the concept that “atypical” features associate more frequently with the presence of NSA-Ab recur in different studies (for a review see [88]).

Indeed, Prüss et al. [11] described the presence of NMDAR-Ab of the IgA isotype in a small cohort of patients with atypical dementia. A subgroup of positive patients partially responded to immunotherapy [11]. Purified IgA containing NMDAR IgA antibodies caused substantial loss of NMDARs and further synaptic proteins in primary hippocampal cultures, resulting in marked changes of NMDAR-mediated currents [11]. These results were further explored in a large series of 660 cases including different neurological disorders and controls. Serum NMDAR-Ab of IgM, IgA, or IgG subtypes were detected in 16.1% of 286 dementia patients and in 2.8% of 217 cognitively healthy controls. Higher prevalence of serum antibodies was detected in patients with “unclassified dementia” followed by PSP, CBD, PD-related dementia (PDD), and primary progressive aphasia (PPA). Among the unclassified dementia group, 60% of 20 patients had NMDAR-Ab, accompanied by higher frequency of CSF abnormalities, and subacute or fluctuating disease progression. Immunotherapy in selected prospective cases resulted in improvement of clinical and functional imaging parameters, as well as antibody disappearance. Epitope mapping showed varied determinants in patients with NMDAR IgA-associated cognitive decline. However, antibodies were rarely found in CSF [12], and therefore their role in the clinical manifestations is unclear. Another study found increased frequency of IgA/IgM serum NMDAR antibodies in patients with atypical AD (i.e., with minimal or no hippocampal atrophy or normal CSF Tau and amyloid β) [13].

In PD, an initial study involving 258 PD patients and 1730 controls did not find an association with the presence of NSA-Ab, although detailed clinical information was not available [89]. In the already discussed Doss et al., study [12], however, NMDAR-Ab, although not overall more frequent in patients with PD, appeared to be more common in PDD than PD without dementia patients [12]. Nevertheless, in a further cohort of PD patients with and without cognitive impairment (*n* = 296) and controls (*n* = 295), the association between NMDAR IgA/IgM antibodies and the presence and severity of cognitive impairment in PD was not confirmed. Moreover, unexpectedly, the frequency of NMDAR IgA/IgM antibodies was lower in PD (13%) than in controls (22%), and positively correlated with age in the latter [90].

Although the appearance of antibodies in neurodegenerative disorders is likely to be secondary, a role in modulating the phenotype has not been systemically investigated. Doss et al. [12] found no association between NMDAR-Abs and neuropsychiatric features in patients with dementia, whereas these antibodies have been associated with minimal symptomatic response to cholinesterase inhibitor treatment, more pronounced cognitive decline and overall poorer prognosis in patients with “non-classical” AD [13].

Antibodies against brain proteins were found in healthy individuals [89,91] as well as in patients with several neurological or psychiatric disorders (reviewed in [92,93]. Whereas the physiological role of some natural occurring antibodies is partially understood (i.e., cellular debris removal) [94], for antibodies against specific neuronal antigens this is unknown, nor are the possible circumstances and mechanisms that may turn these potentially ‘harmless’ antibodies into disease-relevant ones. Dahm L et al. [89] found no difference in immunoglobulin classes distribution (IgM, IgG and IgA) or antibody titres between patients and controls. Therefore, the role of the blood-brain barrier (BBB) in defining the disease status was investigated. It was found that schizophrenic individuals with a past or present history of BBB disturbance were more likely to have more pronounced neurological symptoms if NMDAR antibody seropositive [95]. 

An interesting possibility is that the difference between health- or disease-status could be related to different functionality or epitope specificity of the antibodies. Castillo-Gómez E et al. [96] showed that all NMDAR-Ab positive sera, derived from randomly selected individuals, were able to induce NMDAR internalisation in inducible pluripotent stem cell (IPSC)-derived human cortical neurons. Several different epitopes recognised by NMDAR-Abs were identified, without any consistent functional or epitope pattern related to health/disease state. However, these findings were contradicted by a subsequent study showing different functional effects of NMDAR-Abs from schizophrenic patients versus those found in healthy individuals [97]. NMDAR-Ab from patients, but not from healthy subjects, were shown to alter the surface dynamics and nanoscale organization of synaptic NMDAR and its anchoring partner the ephrin-B2 receptor in heterologous cells, cultured neurons and in mouse brain and prevent long-term potentiation at glutamatergic synapses, while leaving NMDAR-mediated calcium influx intact.

It is clear that more studies are needed to clarify the role of the antibodies in health and disease, mainly to identify the factors associated with the disease status, looking not only at the antibodies per se but also at all those factors that could influence their ability to reach their targets (i.e., BBB integrity) and those which could modulate the immune response (i.e., HLA status). The implications of these findings could help support the use of immunotherapy in patients that otherwise would have remained untreated and avoid ineffective and potentially harmful treatment in those cases which would not benefit. 

## 4. Secondary Neurodegeneration in Patients with Autoimmune Encephalitis

**Figure 2:**
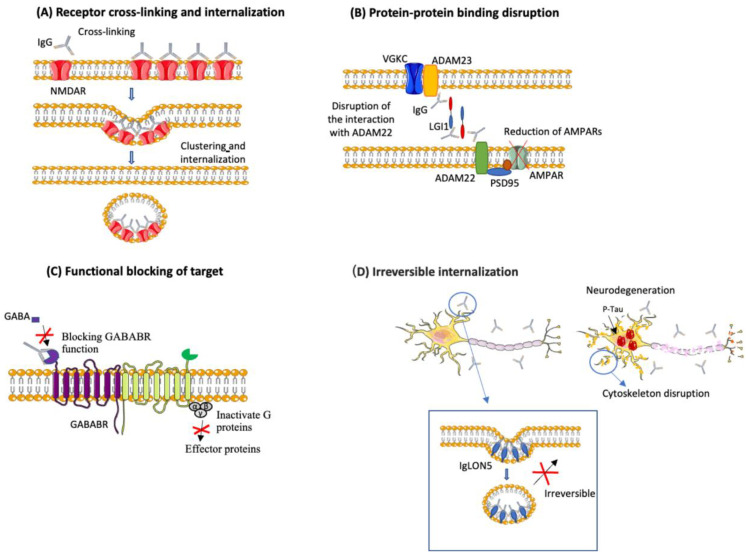
Pathogenic mechanisms of selected NSA-Ab. NSA-Ab alter their target through different mechanisms. (**A**) Some, such as NMDAR-Ab and AMPAR-Ab cause internalization of their targets. (**B**) others (i.e., LGI1-Ab) alter the interaction between their target and partners proteins; (**C**) others (i.e., GABABR-Ab) block their targets. These effects are usually reversible upon removal of the antibody. (**D**) IgLON5 antibodies cause irreversible internalization of IgLON5, causing alterations of the neuronal cytoskeleton, tau accumulation and neurodegeneration.

NSA-Abs disrupt the function of their target in different ways, including internalization, complement activation and through the loss of interaction with other proteins (see Figure 2) [98]. 

In most cases, both animal and in vitro studies demonstrated that the antibody effects are reversible upon antibody removal and indeed patients with AE mediated by NSA-Ab significantly improve with immunotherapy. Nonetheless, is becoming increasingly evident that despite the clinical improvement, a variable proportion of patients present long-term sequelae and do not go back to their pre-morbid condition in terms of cognitive status, employment, and quality of life [21,99]. These observations are supported by changes in long-term cognitive function and brain anatomy as well as changes of neurodegeneration biomarkers, as discussed below.

### 4.1. Long-Term Cognitive Outcome

Long-term cognitive impairment is an increasingly recognized sequela of AE associated with NSA-Ab (Table 2).

In NMDAR encephalitis, cognitive dysfunction is prominent in the acute phase, affecting all domains [107,108]. Memory deficits are the most encountered, followed by executive dysfunction, language impairment and visuospatial difficulties [107]. In the post-acute phase, many patients with NMDAR encephalitis report self-observed deficits including memory problems, concentration difficulties, and social withdrawal [100,102,109]. Several studies have consistently shown persistent cognitive deficits in patients with previous NMDAR encephalitis. A recent systematic review outlined that more than 75% of patients have neuropsychological deficits at any point after treatment, affecting mainly memory and executive function [108]. In most cases, the dysfunction remains stable or improves, although up to 25% of patients may fluctuate or deteriorate over time [108]. As for the general clinical outcome [10,16,110], a worst long-term cognitive function is related to a delay in treatment [100]. Indeed, patients treated more than 3 months after onset have almost eight times the odds of an adverse neuropsychological outcome, compared to those treated earlier [108]. These data were confirmed in a recent study [111] showing persistent cognitive deficits in more than 80% of patients, which was severe in 50%, 2.3 years after onset. At subsequent follow up, 4.9 years after onset, most patients had improved but 30% continued to have severe deficits, mainly involving memory and executive function and few patients had deteriorated, despite the absence of relapse. A poor cognitive long-term outcome was predicted by delayed treatment, older age, and higher disease severity, and longer overall disease duration. The cognitive deficits were not mirrored by functional assessment as measured by the modified Rankin scale (mRS) which showed no deficit in 47.1% of cases, pointing out the importance of neuropsychological assessment in the evaluation of the outcome in these patients. Importantly, this study showed that although cognitive improvement occurs mostly in the immediate post-acute phase, it continues over time, even years after onset [103].

In LGI1 encephalitis, cognitive impairment or encephalopathy are frequent in the acute phase and can persist over time [21,112]. Van Sonderen and colleagues reported persistent amnesia for the disease period and retrograde amnesia in 86% of 21 patients observed over a median follow up period of 42 months [22]. In another study, 29% of patients had moderate to severe cognitive impairment at 24 months follow up, but only 35% were able to return to work or to all premorbid activities [21]. A more in-depth characterization of the associated cognitive impairment showed episodic verbal and visuospatial memory deficits, impaired working memory, impairment of executive function and attention as well as impaired semantic and phonemic fluency [104,113]. As in NMDAR encephalitis, higher disease severity, delays to immunotherapy or longer immunotherapy courses were associated with more severe cognitive dysfunction [104].

Data on long-term cognitive outcome are very scarce in patients with CASPR2 encephalitis. In a study pooling LGI1- and CASPR2-Ab positive cases, some form of cognitive impairment was detected in 28% of patients, with 9% showing a severe dementia at last follow-up using the clinical dementia rating scale (CDR ≥ 0.5) [60]. In another study, including patients with CASPR2-Abs in the CSF, 19% exhibited persistent amnesia at 2 years follow-up [24], although formal neuropsychological testing were not performed. In a small study, assessing memory performance in four patients with CASPR2-Abs at an average of 4.2 (range 3–5) months after immunoadsorption, no memory recovery was observed, despite an overall clinical improvement [114]. However, the observation time was too short and the sample too small to achieve definitive conclusions. Therefore, more data are needed to establish the long-term cognitive outcome of CASPR2 encephalitis.

Similarly, few data are available for other, rarer, forms of AE. In a retrospective study on 22 patients with paraneoplastic GABABR encephalitis, severe anterograde amnesia was observed in all surviving patients at 2-year follow up [105]. In a more recent prospective study, cognitive deficits were detected in 57.1% of patients by CRS and 50% by Montreal cognitive assessment (MoCA) 24 months after onset. Neuropsychological testing revealed deficits in working memory, visual memory, executive function and nonverbal reasoning [106]. Only an older age at onset (≥45 years) was predictive of poor cognitive outcome in this study [106]. In AMPAR encephalitis [33] and DPPX encephalitis [39] 57% and 22% of patients, respectively, had persistent cognitive impairment, although no specific testing was performed. Although cognitive impairment is often overlooked in patients with GlyR-Abs, a recent study found evidence of mild cognitive impairment or dementia in five patients, with prevalent memory impairment, [44] suggesting the need to better explore the cognitive function in patients with GlyR-Ab. No study to date assessed the long-term outcome of GABAAR encephalitis, although in a case series most patients showed a good recovery despite the frequent presence of cognitive impairment at onset [37].

### 4.2. Brain MRI Changes

The long-term cognitive alterations observed in patients with AE are mirrored by permanent structural brain changes. Finke and colleagues [101] first described functional and microstructural alterations in patients with previous NMDAR encephalitis. Using resting state fMRI, they showed reduced bilateral functional connectivity between the hippocampus and the anterior default mode network (aDMN). These alterations correlated with memory performance. Diffusion tensor imaging (DTI) analysis showed widespread white matter changes, which correlated with mRS [101]. Patients with persistent cognitive impairment showed extensive damage of the superficial white matter (WM) including U-fibres and intracortical myelin [115]. On the contrary, grey matter (GM) and routine clinical MRI were normal, despite a tendency towards smaller hippocampal volumes in patients compared to controls [101]. Indeed, in a subsequent study, the same group, using a multimodal imaging approach that combined analysis of hippocampal subfield volumes with DTI derived assessment of hippocampal microstructural integrity, observed hippocampal subfield atrophy and impaired microstructural integrity of the hippocampus in patients recovering from NMDAR encephalitis [116]. Hippocampal volume and microstructural changes correlated with memory performance and disease severity and duration [116]. However, brain structural and functional abnormalities in NMDAR encephalitis seems to extend beyond hippocampus. A number of recent works using different MR techniques as DTI, diffusion kurtosis imaging, voxel-based morphometry (VBM) and surface-based morphometry disclosed diffuse structural changes in hemispheric WM and cortical and subcortical GM, with correlation with cognitive scores (Table 3) [117,118,119,120,121]. Accordingly, network analysis of resting-state fMRI data in patients with post-acute NMDAR encephalitis revealed alterations in whole-brain functional connectivity including impairment of the connectivity between the hippocampus and the medial temporal lobe (MTL) and the DMN, and significant reductions in intranetwork connectivity within the MTL, sensorimotor, lateral-temporal, and visual networks [122]. The disruption of the hippocampal and medial-temporal-lobe network connectivity correlated with memory impairment, whereas schizophrenia-like symptoms were associated with functional connectivity changes in functional connectivity of the frontoparietal control network and the ventral attention network [122].

Long term structural brain changes have also been described in patients with encephalitis previously attributed to VGKC antibodies. In LGI1 encephalitis, follow-up routine MRI showed hippocampal atrophy on visual inspection in most patients, ranging from 77% to 96% of cases [104,113,127]. Volumetric analysis group revealed reductions of all hippocampal subfield volumes except CA1 and mean diffusivity (MD) studies showed altered microstructural integrity. These alterations correlated with memory deficits [104]. Further studies have reported rather conflicting results. Miller and colleagues [124] reported a hippocampal volume loss confined to bilateral CA3, and associated with severe episodic but not semantic amnesia. On the contrary, Hanert and colleagues [113] disclosed a global hippocampal volume reduction in patients with a median time of 3.5 years from the onset of LGI1 encephalitis, associated with deficits in pattern separation performance best predicted by the volume of the hippocampal dentate gyrus, whereas CA1 volume was highly predictive of recognition memory deficits [113]. Regarding extra-hippocampal involvement, Szots and colleagues [128] studied a small sample of patients and reported widespread DTI changes in the cerebral and cerebellar WM, most prominent in the anterior parts of the corona radiata, capsula interna and corpus callosum. Furthermore, MR spectroscopy revealed lower glutamine/glutamate at WM levels and volumetric analysis significantly smaller volume in several brain areas (i.e., cerebellum, brainstem, accumbens, hippocampus, corpus callosum, cerebral WM) [128]. These widespread alterations were not confirmed on a larger sample in which VBM analysis revealed no GM volume change other than in hippocampus and there was no evidence of structural WM damage as assessed using DTI [126]. Notwithstanding, functional connectivity alterations in several large-scale networks, including the DMN which showed an aberrant structure-function relationship with the damaged hippocampus, were evident. In addition, connectivity in the sensorimotor, salience and higher visual networks was impaired independent of hippocampal damage [126].

For encephalitis associated with other type of NSA-Ab, as CASPR2, GABAAR, GABABR and AMPAR, imaging studies focused on follow-up findings are not available except on very small groups or case reports [38,123,129,130,131], generally reporting bilateral mediotemporal abnormalities with possible and variable extra hippocampal involvement, but more in-depth studies are needed for a better characterization. 

### 4.3. Biomarkers and Predictors of Neurodegeneration

Fluid biomarkers of neurodegeneration in AE have been the subject of several studies, especially in the last few years (Table 4). The large majority refers to the CSF and the two most extensively investigated analytes are neurofilament light chain (NFL) and total tau (T-tau). NFL is a component of the cytoskeletal scaffold of large myelinated axons and its CSF concentrations are increased as a consequence of neuroaxonal damage [132]; tau is likewise a mainly axonal protein and is considered a marker of neuronal injury [133].

Increased CSF levels of T-tau and NFL have been reported in AE [135,136] and specifically both in NMDAR [135] and in LGI1 encephalitides [140]. Indeed, the two CSF biomarkers correlate with each other in AE [143]. On the other hand, while NFL levels are similar to those observed in AD [140], T-tau in AE is generally lower than in AD and the more AD-specific biomarker phosphorylated tau (P-tau181) is in most cases not increased [140,144]. Actually, in the large study of Day et al., on 45 cases of NSA-Ab positive AE (NSA-AE; NMDAR, LGI1 and CASPR2), after correction for age CSF levels of T-tau did not significantly differ from those of neurologically healthy controls (HC) [137]. NFL has also been evaluated in serum, where its levels moderately correlate with those in the CSF [145]. Increased levels of NFL in both serum and plasma have been reported in AEs, including NSA-AEs and specifically NMDAR encephalitis compared to HC [138,145,146]. Serum NFL levels in NMDAR encephalitis, however, are lower compared to herpes simplex encephalitis (HSE) [138].

CSF levels of NFL have been reported to be lower in NSA-AEs compared to AEs with antibodies against intracellular antigens (ICAs), while for CSF T-tau the difference was not statistically significant [143]. Conflicting data exist, however, regarding specific forms of NSA-AE, as, for example, some investigations found lower CSF and/or serum levels of NFL in NMDAR encephalitis [142,145], whereas another group observed increased CSF NFL levels in this form but not in LGI1/CASPR2 encephalitis [137]. Several investigations (actually including both NSA-AEs and AEs with antibodies against ICAs) have found higher CSF levels of NFL, but not of T-tau, in patients with neoplasia compared to those without [136,139], although the study of Day et al., on NSA-AEs reported lower CSF NFL levels in the presence of a tumour [137]. While in NMDAR encephalitis an association between CSF NFL and the presence of ovarian teratoma has not been established [147], forms of NMDAR encephalitis developing after HSE or in association with other tumours or demyelinating diseases seem to display higher CSF NFL levels compared to idiopathic or teratoma-associated cases [142].

Several associations between biomarker levels and clinical phenotype have been proposed in AE, albeit not by all investigators [145]. In a cohort including both NSA-AEs and AEs with antibodies against ICAs, CSF NFL correlated negatively with scores on both mini mental state examination (MMSE) and frontal assessment battery (FAB) [143]. In LGI1 encephalitis, CSF levels of NFL seem to be higher in patients with seizures (either clinical or electrographic), but they are not associated with facio-brachial dystonic seizures (FBDSs) specifically [140]. In NMDAR encephalitis, higher CSF levels of NFL have been reported to be associated with extreme delta brush (EDB) on EEG [147], involuntary movements [142], occurrence of prodromal symptoms, seizures or status epilepticus (SE), admission to intensive care unit (ICU), absence of immunotherapy in the first 4 weeks of disease, and subsequent need of second-line immunosuppressive treatment, while for serum NFL levels the only significant associations were those with ICU admission and initial absence of immunotherapy [138].

NFL might correlate with disability, especially longitudinally. In two studies including both NSA-AEs and other AEs, initial CSF levels of NFL, but not of T-tau, correlated with disability (expressed as score on the modified Rankin Scale, mRS) at last follow-up (namely, after 12 months) [135,136]. Similar findings were confirmed for NMDAR and LGI1 encephalitides [142,148], although not in all cohorts [138,140], as well as for serum NFL in NMDAR encephalitis, with the biomarker discriminating between patients with mRS score above vs. below 2 at 1-year follow-up with an area under the curve (AUC) of 0.697 [141]. In agreement with this, serum NFL is lower in NMDAR encephalitis patients with good treatment response compared to those with poor response [141].

Associations between biomarkers and neuroimaging features have been described. In NSA-AEs, Körtvelyessy et al. [139] found higher CSF levels of T-tau and NFL in association with temporal FLAIR hyperintensities on MRI followed, in a later phase, by hippocampal sclerosis. All patients developing hippocampal sclerosis had increased levels of at least one of the two biomarkers, with T-tau seeming to be more predictive of hippocampal sclerosis [139]. In NMDAR and LGI1 encephalitides, higher CSF NFL levels have been reported in patients with abnormal MRI [142], while an association between increased CSF NFL and hippocampal atrophy has been shown in NMDAR encephalitis [147]. Other investigations, however, did not found significant associations with neuroimaging [140,145]. In some studies, biomarkers have been reported to be associated with other laboratory data. In NMDAR encephalitis, CSF levels have been shown to be positively associated with the intensity of antibody positivity in serum [142] and to be higher in patients with CSF pleocytosis [138]. In LGI1 encephalitis, CSF NFL levels are higher in the presence of abnormal CSF and hyponatremia [142].

Regarding potential diagnostic utility of T-tau and NFL in AEs, the most relevant aspects pertain to the differentiation from CJD and psychiatric conditions. CSF levels of T-tau are lower in encephalitis with antibodies against voltage-gated potassium channels (VGKC), LGI1 and other NSAs compared to CJD and discriminate well between NSA-AEs and CJD [134,140,149]. CSF levels of NFL and P-tau181 are lower in LGI1 encephalitis compared to CJD as well [140].

Constantinescu et al. evaluated longitudinal kinetics of CSF NFL and T-tau in a group of 25 patients with AEs (including, but not limited to, NSA-AEs). While NFL levels were in most cases still high at 3-month follow-up and were normalized in one-third of cases at 1-year follow-up, T-tau exhibited faster kinetics, displaying lower levels at 3-month follow-up compared to presentation and being normalized in almost all cases at 1-year follow-up [135]. While in NMDAR encephalitis CSF levels of NFL have been shown to decrease over the disease course in the majority of cases, in LGI1 encephalitis more heterogeneity has been observed, including relatively stable levels [140,142]. Pertaining to T-tau and P-tau181, the absence of significant longitudinal modifications has been reported for CSF levels in LGI1 encephalitis [140]. An investigation performed on patients with NMDAR encephalitis with at least three CSF samplings over the disease course indicated that CSF levels of NFL peaked at a median time of 2.5 months, namely relatively late compared to CSF antibody peak, clinical nadir, and reduction of CSF inflammatory indices (cell count and intrathecal IgG synthesis) [147]. Interestingly, in NMDAR encephalitis, CSF levels of NFL at follow-up have been reported to correlate with mRS scores from the same timepoint, and the same applies to the corresponding differentials of the two measures relative to clinical onset [148]. Moreover, in NSA-AEs the evolution of serum NFL levels seems to mirror the clinical course, with stable levels in clinically stable patients, increasing levels in cases with clinical worsening, and decreasing levels in patients with clinical improvement [145]. Another small study on AEs, not limited to NSA-AEs, reported longitudinally decreasing plasma levels of NFL; accordingly, patients with chronic AE (i.e., evaluated at least 6 months after symptom onset or last clinical deterioration) had lower plasma NFL levels, albeit non-significantly, compared to those with active AE [146]. Although in a study on LGI1 encephalitis no significant difference was observed between levels of T-tau, P-tau181 and NFL from CSF samples taken before or after initiation of immunosuppressive treatment [140], in NSA-AEs reduced CSF levels of T-tau have been reported following immunosuppressive treatment, while the normalization of CSF NFL levels paralleled the resolution of MRI abnormalities [139]. In NMDAR and LGI1 encephalitides, plasma exchange alone or in combination with intravenous immune globulin (IVIG) has been shown to reduce CSF levels of NFL to a greater extent than IVIG alone [142].

As in other neurological diseases, also in AE the phosphorylated form of neurofilament heavy chain (pNFH) has been less extensively studied compared to NFL. In a cohort of AEs, including several cases without antibodies against NSAs, CSF levels of pNFH were higher compared to neurologically healthy controls, were higher in patients who died, but did not differ between cases with vs. without paraneoplastic aetiology and with vs. without antibodies [150]. In NMDAR encephalitis, CSF levels of pNFH have been reported to be lower compared to non-inflammatory neurological controls, without, however, correlating with disability [148]. Similarly, few studies have investigated CSF levels of amyloid β (Aβ) in AE. Although one study on AE patients (of whom not all had NSA-Ab) found a median CSF level of Aβ42 below the cut-off for AD [134], another investigation on AEs with antibodies (including some against ICAs) demonstrated lower CSF Aβ42 and lower CSF Aβ42/Aβ40 ratio compared to AD patients [144]. In a study on LGI1 encephalitis, almost one-third of patients had CSF levels of Aβ42 below the cut-off for AD; however, median CSF Aβ42 was higher compared to AD and similar to CJD and psychiatric patients, and no patient with LGI1 encephalitis had a true CSF AD profile (i.e., reduced Aβ42 along with increased T-tau and P-tau181) [140]. In the same study, baseline CSF levels of Aβ42 correlated positively with MMSE score at 6- and 12-month follow-up, while CSF Aβ42 was longitudinally stable over the disease course [140].

Glial fibrillary acidic protein (GFAP) is a biomarker of astrocytosis and has been investigated by few studies in AEs up to now. In a cohort composed of paraneoplastic neurological syndromes (most of which with brain involvement) and non-paraneoplastic AEs, CSF levels of GFAP were not increased and did not correlate with disability at last follow-up [136]. In a longitudinal study on a similar cohort, GFAP in the CSF tended to increase initially and then to decrease, but the differences between the three timepoints were not statistically significant; equally non-significant was the correlation between CSF levels of GFAP and disability at the time of CSF sampling after correction for age [135]. On the other hand, CSF levels of the microglial (but also astrocytic) biomarker YKL-40 were found to be increased in NSA-AEs compared to neurologically healthy controls [137] as well as in NMDAR encephalitis compared to patients with viral meningitis [151]. In NSA-AEs, CSF levels of YKL-40 correlated with mRS score at 12-month follow-up [137], while in NMDAR encephalitis its levels were diminished at 3-month follow-up, with the extent of decrease correlating with that of mRS score decrease over the same interval [151].

Day et al. also evaluated the less investigated visinin-like protein 1 (VILIP-1, a biomarker of neuronal injury, as the protein is mainly localized in the neuronal cell body) and synaptic biomarkers synaptosome-associated protein of 25 kDa (SNAP-25) and neurogranin in a cohort of 45 NSA-AE cases (NMDAR, LGI1, CASPR2) [137]. Interestingly, all three biomarkers were reduced in AEs compared to neurologically healthy controls, with VILIP-1 also being lower in NMDAR encephalitis than in LGI1/CASPR2 encephalitis. Both VILIP-1 and SNAP-25 were inversely associated with worse mRS scores, ICU admission and presence of a tumour (with the latter two conditions being more frequently observed in NMDAR encephalitis); on the other hand, both SNAP-25 and neurogranin were positively associated with mRS score at 12-month follow-up. Pertaining to the relationships of the synaptic biomarkers, the authors interpreted the early inverse associations with clinical severity as expression of antibody-induced internalization of synapses, and the late direct associations as expression, on the contrary, of loss of synapse integrity [137].

Finally, a recent German multicentre study investigated neurochemical biomarkers in a large cohort of patients with IgLON5 disease (N = 53) [46]. Only 1/25, 3/20, 2/27, and 1/16 patients had high CSF levels of T-tau, high levels of P-tau181, low levels of Aβ42, and low values of the Aβ42/Aβ40 ratio, respectively, with no patients displaying a typical CSF AD profile (i.e., both increased P-tau181 and decreased Aβ42). In 27 patients who had not yet undergone immunosuppressive treatment, serum NFL and GFAP correlated moderately with each other and negatively with CSF cell count. Serum levels of NFL also correlated moderately with CSF T-tau, while serum GFAP was lower in patients with subacute symptom onset and paucisymptomatic phenotype. Moreover, low pre-treatment serum NFL levels independently predicted response to long-term immunotherapy [46].

### 4.4. Mechanisms of Neurodegeneration in AE

**Figure 3:**
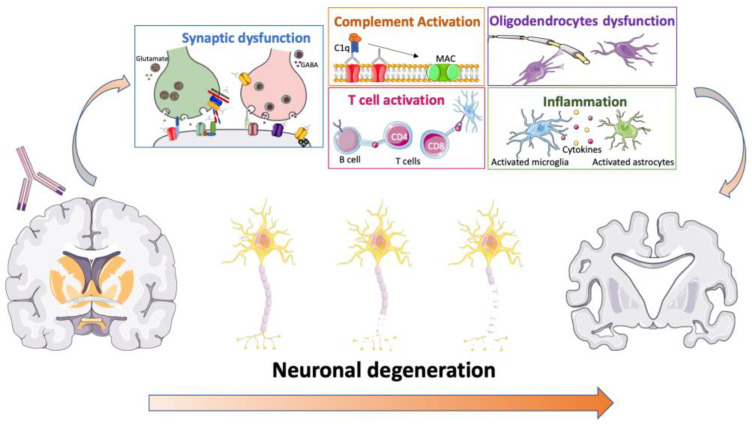
Possible mechanisms of NSA-Ab-mediated neurodegeneration. NSA-Ab can induce synaptic dysfunction, complement activation, T cell, microglia and astrocyte activation and neuroinflammation as well as oligodendrocyte dysfunction. Overall, these alterations might lead to neuroaxonal or neuronal damage with progressive neuronal degeneration and loss.

Although most of the mechanisms underlying NSA-Ab pathogenicity appear to have been elucidated [98], less is known about the mechanisms leading to neurodegeneration in patients with AE (Figure 3). On the contrary, the data available so far from in vitro and in vivo studies seems to be at least partially in contrast to what is observed in patients.

NMDAR antibodies significantly decrease the NMDAR surface and total cluster densities in a titre-dependent fashion with consequent reduction of synaptic NMDAR currents but without disruption of the neuronal structure. The reduction of NMDAR expression is induced by cross-linking and internalization, without complement deposition [17]. In parallel, the antibodies alter the NMDARs trafficking inducing dispersal of GluN2A-NMDAR through the blockade of the interaction between the extracellular domains of GluN1/2 subunits and Ephrin-B2 receptors (EPHB2R) [152]. Altogether these changes affect long term potentiation, which is the cellular substrate of learning and memory, explaining the cognitive symptoms observed in patients. Indeed, the acute administration of NMDAR antagonists, such as ketamine and phencyclidine, induces memory impairment, as well as hallucinations, delusions, agitation and dissociation [153], overall partially reminiscent of NMDAR encephalitis. Similarly, endogenous NMDAR antagonists, including kynurenic acid, endocannabinoids and zinc, among others, have been implicated in the pathogenesis of several neuropsychiatric disorders [154], and particularly schizophrenia [155]. In both in vitro and in vivo models, the effects of NMDAR antibodies on the receptor are reversible. Mice infused with pooled CSFs from individuals with anti-NMDAR encephalitis over 14 days showed memory deficits, anhedonia and depression-like behaviour associated with IgG deposition and a decrease in NMDAR clusters in the hippocampi, which resolved within days after discontinuing the infusion [156]. However, the demonstration of long-term cognitive deficit and structural hippocampal damage suggests that while the acute effects of NMDAR-Abs are functional and reversible, their persistence can impact on neuronal function in a way that goes beyond the established mechanisms and not yet understood. NMDAR is not expressed only on neurons but also on oligodendrocytes where they control the supply of energy substrates to support the axonal functioning via GLUT1 translocation to the oligodendrocyte membrane [157]. Patients’ CSF antibodies reduce the levels of oligodendroglial GLUT1 [158] providing a link with the white matter alterations described in patients with NMDAR encephalitis. It has been shown that the prolonged loss of NMDAR function in oligodendroglia leads to axonal pathology [157] explaining the correlation between disease duration and white matter damage and, indeed patients not recovering from NMDAR encephalitis showed extensive superficial white matter damage [115]. Therefore, it is possible that while neurons recover, the damage to the oligodendrocyte might become permanent, affecting whole brain function. The effects of the antibodies depend on the duration of the exposure. Indeed, in experimental animal studies, NMDAR-antagonists are able to produce reversible or irreversible effects depending on the dosage and duration of the administration [153]. Another, not mutually exclusive possibility, is that the long-term damage is related to the accumulation of excitatory mediators such as glutamate at synaptic levels. Indeed, patients’ CSF and purified IgGs resulted in an acute increase of the glutamate levels of the motor cortex in a rat model [18]. Moreover, the involvement of T cell mediated mechanisms have not been investigated and cannot be derived by in vitro or passive transfer animal models but their possible role has been recently pointed out in an active immunization model [159]. In the few available neuropathological studies, T cell infiltrates have been reported although they usually do not display a cytotoxic phenotype and neuronal degeneration is variable whilst co-occurring pathologies might affect the neuropathological features [160].

Oppositely to NMDAR-Ab, LGI1 antibodies are predominantly IgG4. These kind of IgGs are less effective than IgG1 in crosslinking and internalization and are thought to affect their targets by interference with protein-protein interactions. LGI1-Abs disrupt the ligand-receptor interaction of LGI1 with ADAM22, resulting in reversible reduction in synaptic AMPARs, Kv1.1 and VGKC [23,161]. Nevertheless, LGI1-Abs have been detected in the sera of cats with a spontaneous form of autoimmune encephalitis with complex partial seizures with orofacial involvement. Post-mortem analysis in these cases showed complement deposition, a feature also shared by post-mortem examination of the small number of available brains from patients with LGI1-related encephalitis [162]. The finding of complement deposition suggests that LGI1-Abs can induce neuronal death through the activation of the complement cascade which may explain the persistence of long-term cognitive deficits as well as structural brain changes in affected patients. The post-mortem findings reflect the relatively small, but more pathogenic, IgG1 antibodies that are present in some patients with LGI1 encephalitis and occur more frequently in patients with cognitive impairment [19,20]. The same might be true for CASPR2-Abs. Indeed, CASPR2-Abs are mainly IgG4, and therefore the most likely common mechanism of their action is a disruption of the interaction of CASPR2 with its associated molecules rather than internalization and complement activation. Accordingly, CASPR2-Abs did not reduce CASPR2 expression on the surface of cultured hippocampal neurons, but they appeared to act by altering CASPR2 interaction with contactin-2 [163,164]. However, these findings contrast with the results of others who found CASPR2-Abs mediated internalization of their target [25,26,27] as well as with neuropathology showing reduced CASPR2 expression in the brain [165] neuronal loss and complement deposition [166,167]. These discrepancies might be related to different proportion of IgG1 and IgG4 in the samples used in various studies which might affect the prevalent observed mechanism. Moreover, similarly to LGI1-Abs, CASPR2-Abs may also alter AMPAR synaptic traffic affecting cognitive function at least in the acute phase [26,27].

AMPAR [35,36] and GABABR [31] antibodies act causing internalization and block of the receptor, respectively. However, neuropathological evidence of T cell activation and inflammation [32,168] together with the evidence of hippocampal sclerosis and evolution in brain atrophy suggest the presence of additional mechanisms promoting neurodegeneration.

## 5. IgLON5 Disease: The Interface between Antibody-Mediated Immunity and Neurodegeneration

The question of the relation between antibodies and neurodegeneration is made more complex by the description of IgLON5 antibodies. These were firstly described in a small group of patients with prominent sleep-related movement disorders, mainly characterised by a non-REM sleep parasomnia with simple or finalistic movements, resembling daytime activities such as eating, drinking or manipulating objects. Other sleep abnormalities included RBD and periodic limb movements during sleep. Breathing disorders, including obstructive sleep apnoea (OSAS) and stridor, both in sleep and wakefulness, are almost a constant feature and are often associated with respiratory failure. Other clinical manifestations include ataxia, bulbar signs, abnormal eye movements and dysautonomia [28,45,167,169]. Over time, other clinical manifestations were reported including dementia and chorea [170,171,172], motor neuron-like disease and seizures [147,173]. In the first case series, all eight patients reported received immunotherapy, but six of them died during the follow-up period [45]. To date more than 100 patients have been reported and response to immunotherapy seems to occur in certain cases and to some extent [46,170,171,172,173,174,175]. Initiation of first-line immune treatment within 6 weeks, or the initiation of long-term treatment within 1 year of onset, seems to predict clinical improvement; moreover, a low serum level of neurofilament light chain at onset correlated with a better prognosis [46]. However, mortality remains high [46,175,176]. Neuropathology, intriguingly, show the presence of hyperphosphorylated three- and four-repeat tau aggregates in neurons, and neuronal loss predominantly in the hypothalamus and the brainstem tegmentum, and absence of inflammatory infiltrates [45,177]. However, not all patients show these neuropathological features. Some cases showed no p-tau deposition [173,178] or no deposition in the brainstem [179,180], together with evidence of neuroinflammation, including lymphocyte infiltration and microglia activation [173,178,179,180]. These findings, together with the finding of increased B cell numbers in the CSF [181], suggest that neurodegeneration might be a secondary disease mechanism, that set after an initial immune response, which would fit with the observed response to immunotherapy in patients treated close to disease onset. Moreover, all genotyped patients had HLA-DQB1*0501 and HLA-DRB1*1001 alleles, suggesting a genetic susceptibility for autoimmunity [45].

The IgLON5 antibodies target the extracellular domain of the protein and are predominantly of the IgG4 subtype, and to a lesser extent IgG1. The latter are responsible for the irreversible internalisation of IgLON5 in rat neuronal cultures [182], whereas this effect was not seen with IgG4 antibodies, which are likely to act in a different way. Another study, demonstrated that IgLON5 antibodies disrupt the cytoskeleton in rat hippocampal neurons, resulting in dystrophic axons and axon swelling after 3 weeks (Figure 2D). These alterations might explain the raised concentration of NFL in patients’ CSF [183]. Ryding et al. [184], showed progressive p-tau accumulation, synaptic disruption, neuronal death as well as reduction in IgLON5 concentrations, in differentiated human neural stem cells incubated with IgLON5 antibodies [184]. These pathogenetic effects of IgLON5 antibodies have been confirmed in vivo [47,185]. Mice infused with these antibodies showed p-tau deposition in the hippocampal CA4 region, mossy fibres, and posterior periependymal areas [185]. In another study, mice exposed to IgLON5 antibodies showed neuronal loss, microglial and astrocytic activation as well as an increase in the relative mRNA expression levels of several inflammatory factors, including TGF-β, CCL5, and CXCL13 [47]. Altogether, these observations suggest that IgLON5 antibodies are directly pathogenic and can initiate an inflammatory and neurodegenerative process. However, the differential role of IgG1 and IgG4 in these process remains to be clarified, as well as the contribution of adaptive immunity and neuroinflammation in the progression of the disease.

## 6. Conclusions

Several NSA-Ab can present with cognitive impairment, phenocopying different neurodegenerative disorders. These antibodies are pathogenic in this context and the unusual presentation, often associated with the lack of typical inflammatory CSF and imaging findings, might lead to misdiagnosis, and delay the immune treatment. Although few reports have suggested clinical red-flags for this autoimmune dementia, their real utility in predicting this diagnosis needs to be investigated in prospective studies. On the other hand, NSA-Ab have been detected in patients with established neurogenerative disorders; in these non-encephalitis situations their pathogenic potential is debated and to date there is no evidence on the possible utility of immunotherapy.

In addition to NSA-Ab, several antibodies have been described in patients with different neurodegenerative disorders [8,186,187] and they might have a role in modulating the neurodegenerative process as well as the clinical phenotype. We suggest that a similar role might be also played by NSA-Ab outside of encephalitis. Indeed, these neuronal antibodies have the potential to induce neurodegeneration with permanent cognitive and brain structural changes in patients with AE. Moreover, IgLON5 antibodies suggest a continuum between autoimmunity and neurodegeneration. Indeed, it is possible that multiple antibodies against neuronal proteins either with detrimental or neuroprotective function occur together contributing to the individual risk of developing a neurodegenerative disorder [188]. Therefore, future studies should investigate the pathogenic potential of NSA-Ab in neurodegenerative disorders. A better understanding of the interaction between humoral immunity and neurodegeneration might pose the base for novel therapeutic options in patients with neurodegenerative disorders.

## Figures and Tables

**Table 2:** Representative studies investigating long-term cognitive outcome in patients with AE.

Antibody Target (Ref.)	N. of Patients Gender and Age	Time from Disease Onset (Months)	Frequency of Cognitive Impairment during the Chronic Phase	Features of Cognitive Impairment (Main Affected Domain)	NPS Testing	Predictors of Cognitive Outcome
NMDAR [100]	9 (8 F) mean age 28.4 y, range 21–44	Median 43 m, range 23–69	78% of patients exhibited residual cognitive impairment	Deficits in attention, memory (working and episodic) and executive function	Full NPS assessment ^1^	Better cognitive outcome in patients with early immunotherapy
NMDAR [101]	24 (21 F) mean age 27.9 ± 1.6 y	Median 35, range 9–63	79% of patients exhibited residual cognitive impairment	Deficits in attention, memory (working and episodic) and executive function	Full NPS assessment ^1^	none
NMDAR [102]	7 (6 F), mean age 26.42 ± 8.54 y	Median 19, range 7–34	All patients had subjective cognitive impairment	Verbal/visual memory, working memory, attention, processing speed, executive functioning, and social cognition	Full NPS assessment ^1^	NA
NMDAR [103]	40 (35 F) mean age 28.5 ± 7.2	Median 2.3, (0.3–7.0) years at first visit, 4.9 (2.3–9.4) at second visit	73% of the patients at first and 50% at second study visit reported subjective cognitive complaints	Deficits of verbal episodic memory, working memory, and executive function	Full NPS assessment ^1^	Treatment delay, long hospitalization times, disease severity, need for ICU admission associated with a worse outcome
NMDAR [99]	56 (49 F), 44 at 2 years FU	Median 45, (range, 1–116)	24 (62%) continued to have some sort of sequelae	Memory disorders (16/39, 41%), psychotic symptoms (8/39, 21%), speech disorders (4/39, 10%), 27 (61%) returned to their previous work or school life	Questionnaire	NA
LGI1 [21]	76 (mean age 61 years (range 32–80)), 48 with data at 2-years follow-up	2 years FU	29% had moderate to severe cognitive problems	NA	In-house cognitive performance score	Failure to respond to firs line immunotherapy associated with cognitive impairment
LGI1 [104]	30 (11 F), mean age, 65.7 ± 12.3	Median 23.3 months	NA	Verbal and visuospatial memory deficits compared to controls	Full NPS assessment ^1^	The latency between disease onset and initiation of immunotherapy correlated with verbal and visuospatial memory deficits
LGI1 [22]	38 (13 F) 21 with FU > 2 years	Median 42 months	86% had persistent amnesia for the disease period	Altered spatial recognition	Cambridge Neuropsychological Test Automated Battery (performed in 11 patients)	NA
LGI1 [20]	103 (64 M) median age 64, range 22–92	48 months	78% of patients developed cognitive impairment	NA	MMSE/MoCA, ACE-R	FBDS correlated with cognitive impairment
LGI1/ CASPR2 [60]	95 (89 with outcome)	Median 35 months, range 57–456)	Severe dementia was documented in 9%. However, at last follow-up, 62% (51 of 82) with central involvement had residual cognitive/personality disturbance	NA	CDR	NA
GABABR [105]	22 (4 F) 64 years (range 55–85)	12 months (13 patients), 24 months (6 patients)	At 12 months all had anterograde amnesia, temporal and spatial disorientation and 54% had neurospychiatric symptoms. None recovered to pre-morbid status and occupation	Severe amnestic dysfunction with mild dysexecutive troubles	MMSE, FAB; most had a MoCA or a more extensive neuropsychological test (ADAS-Cog)	NA
GABABR [106]	31 (19 M) median age 52 (18–75)	18 (range: 6–63 months	At 24 months FU, 57.1% (8/14) had CPS > 2 and 50.0% (5/ 10) had MoCA < 26. Eight (80%) patients exhibited cognitive deficits in the cognitive tests. 50% (7/14) had persistent neuropsychiatric symptoms	Impairment in working memory, visual memory, attention, executive functions and nonverbal reasoning	CRS, MoCA, NPI, full NPS ^1^	Age of disease onset ≥45 years was a risk factor for long-term cognitive deficits
AMPAR [33]	7 (4 F), median age 56 y (21–92)	Median 12 m (2–31)	57% had cognitive impairment	Memory disorder	NA	Worst outcome in patients with fulminant encephalitis
DPPX [39]	9 (7 M) median 57 (36–59)	Median 19 (6–72 m)	22% mild cognitive deficits	NA	NA	NA

ACE-R: Addenbrooke’s cognitive examination-revised; ADAS-Cog: Alzheimer’s disease assessment scale-cognitive subscale; CDR: clinical rating scale; CPS: cognitive performance scale; CRS: cognitive rating scale; F: female; FU: follow-up; M: male; MMSE: mini-mental state examination; MoCA: Montreal cognitive assessment; NA: not available; NPI: neuropsychiatric inventory; NPS: neuropsychological; Y: years. ^1^ Digit span forward and backward, block tapping forward and backward, Rey auditory verbal learning test [RAVLT] and Rey-Osterrieth complex figure test, Stroop test, semantic fluency, verbal fluency, tower of London, BADS, premorbid intelligence quotient.

**Table 3:** Representative studies investigating long-term brain imaging changes and their correlation with cognitive outcome in patients with AE.

Antibody Target (Ref.)	N. of Patients Gender and Age	Time from Disease Onset (Months)	Features of Cognitive Impairment (Main Affected Domain)	NPS Testing	MR Technique	MR Features	Correlation with NPS
NMDAR [101]	24 (21 F), mean age 27.9 ± 1.6 years)	Median 35, range 9–63	Deficits in attention, memory (working and episodic) and executive function.	Full NPS assessment ^1^	DTI, VBM, hippocampal volumetry, Rest-fMRI	Reduced functional connectivity of the hippocampus with the aDMN. Extensive WMC, most prominent in the cingulum	Connectivity of both hippocampi predicted memory performance in patients. WMC correlated with disease severity
NMDAR [116]	40 (36 F); mean age, 28.0 ± 1.6 years	Mean 26.6 ± 3.3 months	Verbal and visuospatial memory deficits	RAVLT, ROCF	Hippocampal volumetry and DTI	Reduced hippocampal volumes; bilateral atrophy of the input and output regions of the hippocampal circuit. Impaired microstructural integrity	Hippocampal volumetric and microstructural integrity measures correlated with memory performance
NMDAR [115]	46 (40 F) age, mean 26.67 ± 8.45	27.6 ± 4.6 months after symptom onset	Impairments in working, verbal, visuospatial memory and attention	Full NPS assessment ^1^	DTI, vertex-based analyses	Non-recovered patients showed widespread superficial WM damage. Damage predominated in frontal and temporal lobes	Persistent cognitive impairment correlated with damage of the superficial WM
NMDAR [117]	15 (8 M); age 29.20 ± 11.63	4.41 ± 1.78 months	Patients MoCA lower than controls, especially in executive function, fluency, delayed recall and visual perception items	MoCA	DTI	FA reduction in right MTG, left MCP, right PC, and an MD increase in left medial temporal gyrus and left frontal lobe	The FA value of right PC positively correlated with total MoCA score and fluency score. The MD of left frontal lobe negatively correlated with total MoCA score, and MD of the left MTG positively correlated with delayed recall
NMDAR [118]	57 (27 M); median age 31, IQR 23,46	15.44 ± 9.08 months	Significantly decreased MoCA scores	MoCA	DTI and DKI	Decreased RK in the right extranucleus in WM and notably decreased KFA in the right PC, the right SPG, the left PC, left MOG, and left SOG in GM. GM regions with decreased KFA in the left MTG, STG, SMG, POCG, IPL and ANG	The KFA and RK in the left ANG, IPL and POCG correlated positively with MoCA scores
NMDAR [119]	22 (10 males); mean age 30.54 ± 10.79	46.91 ± 19.59 days	Significantly lower scores in MoCA, Auditory Verbal Learning Test-Immediate Recall (AVLT_IR), and Auditory Verbal Learning Test-Delayed Recall (AVLT_DR)	MoCA; AVLT	VBM, rest-fMRI	Significant GM atrophy in the bilateral triIFG and right PC, decreased connectivity between: left triIFG and bilateral HES, between right triIFG and HES, between right PC and left cerebellum, and increased connectivity between right triIFG and left SFG.	GM volume in right triIFG and decreased connectivity between left triIFG and right HES were associated with decreased memory scores
NMDAR [120]	24 (12 M); mean age 29.41 ± 10.99	Mean 15.16 months	Trend of decreased MMSE and significantly decreased AVLT_IR and AVLT_DR	MMSE and AVLT	Surface-based morphometry, Hippocampal volumetry, and subfield segmentation	Decreased cortical volume mainly in language network and DMN and decreased GM volume in left CA1 body of hippocampus	Decreased cortical thickness in the right SFG associated with decreased cognitive scores; decreased cortical volume in the right pars triangularis and decreased surface area in the right pars opercularis associated with decreased memory scores
NMDAR [121]	40 (16 males; age 27.33 ± 9.31)	Mean 19.55 ± 14.42 months	Significantly lower MoCA scores and significantly higher executive control scores	MoCA, ANT	VBM	Decreased GM volume in bilateral thalamus, left mPFC, left STG, and left rectus gyrus. Lower GM volume in the left PC and right posterior cerebellar lobe in patients with disease duration >2 years	“Executive control score was negatively correlated with GM volume in mPFC and the right thalamus. STG GM volume was positively correlated with MoCA score
VGKC [123]	24 (14 LG1, 4 LGI1/CASPR2, 6 VGKC); (20 male; mean (SD) age 62.36 (12.09) years)	Mean (SD) 5.22 (3.77) years	Patients were impaired on visual and verbal recall and verbal recognition memory measures	Full NPS assessment ^1^	Volumetry, VBM, rest-fMRI	Focal hippocampal atrophy within the medial temporal lobes, correlative atrophy in the mediodorsal thalamus, and additional volume reduction in the posteromedial cortex	There was no association between regional volumes and memory performance. Instead, patients demonstrated reduced posteromedial cortico-hippocampal and interhippocampal connectivity, which correlated with memory scores
LGI1 [104]	30 (11 F), mean age, 65.7 ± 12.3 years	Median, 23.3 months; IQR 6.4–35.4 months	Significant and persisting verbal and visuospatial memory deficits	Full NPS assessment ^1^	DTI, VBM, hippocampal and basal ganglia volumetry	Hippocampal atrophy, including subfields CA2/3 and CA4/DG, as well as impaired hippocampal microstructural integrity. No abnormalities of cortical GM or WM were found	Higher disease severity correlated with larger verbal memory deficits, decreased volumes of left hippocampus and left CA2/3 and CA4/DG subfields, and impaired left hippocampal microstructural integrity. Decreased volume of the left CA2/3 subfield and impaired left hippocampal microstructural integrity correlated with verbal memory deficits
LGI1 [113]	15 (9 M) mean age: 64.47 ± 3.28 years	3.53 ± 0.65 years after onset	Overall memory deficit including a significant reduction in pattern separation performance	Full NPS assessment ^1^	Hippocampal volumetry and subfield segmentation	Global hippocampal volume reduction	Deficits in pattern separation performance were best predicted by the DG, whereas CA1 was highly predictive of recognition memory deficits.
LGI1 [124]	18 (mean age: 64.0 ± 2.55 years; range: 24–71; 15 male)	Median 4 years	Selective and severe loss of internal (episodic) but no loss of external (semantic) autobiographical memory detail in the patients compared to the controls	Full NPS assessment ^1^	Hippocampal volumetry and subfield segmentation, VBM	Hippocampal volume loss confined to bilateral CA3	CA3 subfield atrophy was associated with severe episodic but not semantic amnesia for postmorbid autobiographical events that was predicted by variability in CA3 volume
LGI1 [125]	9 (5 M) mean age 59.9 ± 14.5	33.1 ± 18 months	ACE score 83 at 24 months in 5 patients	MMSE and ACE 24 months after onset	DTI and MRS, volumetric analysis	DTI showed widespread changes in the cerebral and cerebellar WM, most prominent in the anterior parts of the corona radiata, capsula interna and CC. MRS revealed lower glutamine/glutamate WM levels. Significantly smaller volume in several brain areas (i.e., cerebellum, brain stem, accumbens, hippocampus, CC, cerebral WM)	Higher putaminal volume was associated with better cognition by ACE test at 24 months
LGI1 [126]	27 (18 M) mean age 65.8 ± 11.4 years	25.9 ± 16.7 months	Significantly impaired working memory, verbal and visual learning and episodic memory; executive dysfunction and decreased semantic fluency	Full NPS assessment ^1^	DTI, VBM, hippocampal volumetry, Rest-fMRI	Reduced hippocampal volume. Functional connectivity alterations in several large-scale networks, including the DMN. Impaired connectivity in the sensorimotor, salience and higher visual networks	Increased connectivity in ventral and dorsal DMN regions significantly correlated with better memory performance. Stronger connectivity of the insula with the salience network and DMN was linked to impaired memory function

ACE: Addenbrooke’s cognitive examination test; aDMN: anterior default mode network; ANG angular gyrus; ANT: attention network test; AVLT: auditory verbal learning test; AVLT-IR: immediate recall; CA: cornu ammonis; CC: corpus callosum; DR: delayed recall; DKI: diffusion kurtosis imaging; DG: dentate gyrus; DTI: diffusion tensor imaging; FA: fractional anisotropy; FBDS: faciobrachial dystonic seizures; GM: grey matter; HES: Heschl gyrus; IPL: inferior parietal but supramarginal gyrus; IQR: interquartile range; KFA: kurtosis FA; MCP middle cerebellar peduncle; MD: mean diffusivity; MMSE: mini-mental state examination; MoCA: Montreal cognitive assessment; MOG: middle occipital gyrus; mPFC: medial prefrontal cortex; MRS: magnetic resonance spectroscopy; MTG: middle temporal gyrus; PC: praecuneus; POCG: postcentral gyrus; RAVLT: Rey auditory verbal learning test; RK: radial kurtosis; ROCF: Rey-Osterrieth complex figure; SFG: superior frontal gyrus; SMG: supramarginal gyrus; SOG: superior occipital gyrus; SPG: superior parietal gyrus; STG: superior temporal gyrus; triIFG: triangle part of the inferior frontal gyrus; VBM: voxel-based morphometry; WM: white matter; WMC: white matter changes. ^1^ Digit span forward and backward, block tapping forward and backward, Rey auditory verbal learning test [RAVLT] and Rey-Osterrieth complex figure test, Stroop test, semantic fluency, verbal fluency, tower of London, BADS, premorbid intelligence quotient.

**Table 4:** Representative studies investigating neurochemical biomarkers of neurodegeneration in autoimmune encephalitides.

Study	Autoimmune Encephalitis	Other Diagnostic Categories	Biological Fluids	Biomarkers	Main Findings
Chang et al., 2022 [134]	*n* = 15 (with known antibodies, *n* = 8: LGI1, *n* = 2; neuronal intermediate filaments, *n* = 2; NMDAR, *n* = 1; CASPR2, *n* = 1; DPP6, *n* = 1; IgLON5, *n* = 1)	sCJD (*n* = 11)	CSF	Aβ42, Aβ40, Aβ42/Aβ40 ratio, T-tau, P-tau181	In AE, levels of T-tau and P-tau181 are normal The median level of Aβ42 in AE is low (i.e., below the cutoff for AD) T-tau discriminates well between AE and sCJD
Constantinescu et al., Eur J Neurol 2016 [135]	*n* = 25 (with known antibodies, *n* = 12: Ma2, *n* = 2; NMDAR, *n* = 4; GAD, *n* = 5; TPO, *n* = 4, LGI1, *n* = 1; GABABR, *n* = 1)	NA	CSF	NFL, T-tau, GFAP	NFL initially increased in 19/24 patients, still high in most cases at 3-month follow-up, and normalized in one-third of cases at 12-month follow-up T-tau initially increased in 17/22 patients, lower at 3-month follow-up, and in almost cases normal at 12-month follow-up GFAP: no significant differences NFL at all timepoints (initial evaluation, 3-month follow-up and 12-month follow-up) and T-tau at 3-month follow-up and at 12-month follow-up correlate with final disability (mRS score at 12-month follow-up)
Constantinescu et al., J Neuroimmunol 2017 [136]	Paraneoplastic neurological syndromes (in most cases with brain involvement), *n* = 16 (with overt malignancy, *n* = 12) Non-paraneoplastic AE, *n* = 21 (NMDAR, *n* = 3)	NA	CSF	NFL, T-tau, GFAP	NFL and T-tau are increased in both paraneoplastic neurological syndromes and non-paraneoplastic AE. GFAP is not increased NFL is higher in paraneoplastic neurological syndromes compared to non-paraneoplastic AE NFL correlates with disability at last follow-up
Day et al., Neurology 2021 [137]	*n* = 45 (NMDAR, *n* = 34; LGI1, *n* = 7; CASPR2, *n* = 4)	Neurologically healthy controls (*n* = 39)	CSF	T-tau, VILIP-1, YKL-40, SNAP-25, neurogranin	After correction for age, T-tau does not differ between AE and controls NFL is increased in NMDAR AE but not in LGI1/CASPR2 AE YKL-40 is increased in AE SNAP-25 and neurogranin are reduced in AE VILIP-1 is reduced in NMDAR AE compared to LGI1/CASPR2 AE VILIP-1 and SNAP-25 correlate negatively with worst mRS score Higher SNAP-25, neurogranin and YKL-40 levels are associated with higher mRS scores at 12-mont follow-up VILIP-1 and SNAP-25 are lower in patients admitted to ICU and in patients with neoplasia (features which are more common in NMDAR AE) NFL is lower in patients with neoplasia.
Grüter et al., Brain 2022 [46]	IgLON5 disease, *n* = 53	NA	CSF, serum	Aβ42, Aβ40, Aβ42/Aβ40 ratio, T-tau, P-tau181 (CSF) NFL, GFAP (serum)	CSF T-tau is increased in 1/25 patients. CSF P-tau181 is increased in 3/20 patients. Aβ42 is decreased in 2/27 patients. Aβ42/Aβ40 ratio is decreased in 1/16 patients. However, no patient has a typical AD pattern (i.e., decreased Aβ42 and increased P-tau181) sNFL and sGFAP at first evaluation correlate moderately with each other and correlate negatively with CSF cell count sGFAP at first evaluation is lower in patients with subacute onset and with paucisymptomatic phenotype sNFL at first evaluation correlates moderately with CSF T-tau Lower sNFL at first evaluation is an independent predictor of response to long-term immunotherapy
Guasp et al., Neurology 2022 [138]	NMDAR AE, *n* = 118 (patients with isolated psychosis, *n* = 33)	Patients with first episode of psychosis of psychiatric origin, *n* = 45 HSE, *n* = 36 Neurologically healthy controls, *n* = 36	Serum, CSF	NFL	sNFL in NMDAR AE is higher compared to psychiatric patients and controls and lower compared to HSE sNFL discriminates between NMDAR AE with isolated psychosis and psychiatric patients (AUC = 0.93; with a cutoff of 15 pg/mL, Se = 85% and Sp = 96%) In NMDAR AE, higher CSF NFL is associated with prodromal symptoms, seizures/status epilepticus, ICU admission, CSF pleocytosis, absence of immunosuppressive treatment in the first 4 weeks, and subsequent second-line immunosuppressive treatment, while for sNFL the only significant associations are those with ICU admission and absence of immunosuppressive treatment in the first 4 weeks
Körtvelyessy et al., Front Neurol 2018 [139]	*n* = 38 (NMDAR, *n* = 18; LGI1, *n* = 10; CASPR2, *n* = 8; GABABR, *n* = 1; AMPAR, *n* = 1)	Non-neurodegenerative and non-inflammatory neurological controls, *n* = 45	CSF	NFL, T-tau	7/23 patients have high NFL levels. Of these 7, 5 have MRI abnormalities in limbic structures which subside following immunosuppressive treatment; this is paralleled by a normalization in NFL levels High initial levels of NFL and T-tau are associated with temporal FLAIR hyperintensities followed by hippocampal sclerosis in MRI. T-tau is more strongly predictive of hippocampal sclerosis compared to NFL T-tau is reduced after immunosuppressive treatment T-tau is not influenced by presence of neoplasia
Lardeux et al., J Neurol 2022 [140]	LGI1 AE, *n* = 24	sCJD, *n* = 20 Psychiatric patients, *n* = 20 AD, *n* = 39	CSF	NFL, Aβ42, T-tau, P-tau181	8/20 patients with LG1 AE have increased T-tau levels 1/20 patients with LGI1 AE have increased P-tau181 6/19 patients with LGI1 AE have decreased Aβ42. However, no patient has a typical AD profile (i.e., decreased Aβ42 and increased T-tau and P-tau181) In general, the biomarkers in LGI1 AE are longitudinally stable T-tau and P-tau181 in LGI1 AE are similar to psychiatric patients and lower compared to AD and sCJD Aβ42 in LGI1 AE is similar to psychiatric patients and sCJD and higher compared to AD NFL is LGI1 AE is similar to AD, higher than in psychiatric patients and lower than in sCJD In LGI1 AE, NFL is higher in patients with seizures (clinical or EEG) compared to patients without seizures In LGI1 AE, the biomarkers are not associated with FBDS, temporo-mesial T2 hyperintensity on MRI, mRS score, or immunosuppressive treatment In LGI1 AE, Aβ42 correlates with longitudinal MMSE score
Ma et al., Neurol Sci 2022 [141]	NMDAR AE, *n* = 64	Neurologically healthy controls (*n* = 84)	Serum	NFL	In NMDAR AE, NFL is higher than in controls Patients with mRS scores >3 have higher NFL levels than patients with mRS scores ≤3 Patients with good response to treatment have lower NFL levels than patients with poor response to treatment NFL levels correlate with mRS score at 1-year follow-up AUC for the prediction of mRS score at 1-year follow-up (mRS score >2 vs. ≤2): 0.697
Nissen et al., Front Immunol 2021 [142]	NMDAR AE, *n* = 37 (idiopathic/teratoma-associated, *n* = 27; secondary post-HSE, *n* = 5; SCLC and limbic encephalitis with anti-Hu antibodies, *n* = 1; PCNSL (B-cell), *n* = 1; MS, *n* = 2; ADEM; *n* = 1) LGI1 AE, *n* = 16	NA	CSF	NFL	NFL is lower in idiopathic/teratoma-associated NMDAR AE compared to LGI1 AE NFL levels in idiopathic/teratoma-associated NMDAR AE decreased longitudinally, while the course is more heterogeneous in LGI1 AE NFL correlates with final mRS score in idiopathic/teratoma-associated NMDAR AE and LGI1 AE NFL is lower in idiopathic/teratoma-associated NMDAR AE compared to secondary NMDAR AE In idiopathic/teratoma-associated NMDAR AE, NFL is associated with intensity of antibody positivity in serum In idiopathic/teratoma-associated NMDAR AE, higher NFL levels are associated with abnormal MRI, involuntary movements and higher mRS score at last follow-up In LGI1 AE, higher NFL levels are associated with abnormal CSF findings and hyponatremia In NMDAR AE, PLEX alone or combined with IVIG is associated with greater longitudinal decrease of NFL compared to IVIG alone

Abbreviations. AD, Alzheimer’s disease. ADEM, acute disseminated encephalomyelitis. AE, autoimmune encephalitis. AMPAR, alpha-amino-3-hydroxy-5-methyl-4-isoxazolepropionic acid receptor. CASPR2, contactin-associated protein-like 2. sCJD, sporadic Creutzfeldt-Jakob disease. CSF, cerebrospinal fluid. DPP6, dipeptidyl-peptidase-like protein 6. FBDS, facio-brachial dystonic seizures. GABABR, gamma-amino-butyric acid receptor subunit B. GAD, glutamic acid decarboxylase. GFAP, glial fibrillary acidic protein. HSE, herpes simplex encephalitis. ICU, intensive care unit. IVIG, intravenous immune globulin. LGI1, leucine-rich glioma-inactivated 1. Ma2, membrane active protein 2. MMSE, mini mental state examination. MRI, magnetic resonance imaging. mRS, modified Rankin Scale. MS, multiple sclerosis. NFL, neurofilament light chain. NA, not applicable. NMDAR, N-methyl-D-aspartate receptor. PCNSL, primary central nervous system lymphoma. PLEX; plasma exchange. P-tau181, tau phosphorylated at amino acid residue 181. SCLC, small-cell lung cancer. Se, sensitivity. sGFAP, serum glial fibrillary acidic protein. Sp, specificity. SNAP-25, synaptosome-associated protein of 25 kDa. sNFL, serum neurofilament light chain. TPO, thyroid paroxidase. T-tau, total tau. VILIP-1, visinin-like protein 1.

## Data Availability

All data have been included in the manuscript.
